# Nanodiamonds suppress the growth of lithium dendrites

**DOI:** 10.1038/s41467-017-00519-2

**Published:** 2017-08-25

**Authors:** Xin-Bing Cheng, Meng-Qiang Zhao, Chi Chen, Amanda Pentecost, Kathleen Maleski, Tyler Mathis, Xue-Qiang Zhang, Qiang Zhang, Jianjun Jiang, Yury Gogotsi

**Affiliations:** 1 A.J. Drexel Nanomaterials Institute, and Department of Materials Science and Engineering, Drexel University, 3141 Chestnut Street, Philadelphia, PA 19104 USA; 2 Department of Chemical Engineering, Beijing Key Laboratory of Green Chemical Reaction Engineering and Technology, Tsinghua University, Beijing, 100084 People’s Republic of China; 3 School of Optical and Electronic Information, Huazhong University of Science and Technology, Wuhan, Hubei 430074 People’s Republic of China

## Abstract

Lithium metal has been regarded as the future anode material for high-energy-density rechargeable batteries due to its favorable combination of negative electrochemical potential and high theoretical capacity. However, uncontrolled lithium deposition during lithium plating/stripping results in low Coulombic efficiency and severe safety hazards. Herein, we report that nanodiamonds work as an electrolyte additive to co-deposit with lithium ions and produce dendrite-free lithium deposits. First-principles calculations indicate that lithium prefers to adsorb onto nanodiamond surfaces with a low diffusion energy barrier, leading to uniformly deposited lithium arrays. The uniform lithium deposition morphology renders enhanced electrochemical cycling performance. The nanodiamond-modified electrolyte can lead to a stable cycling of lithium | lithium symmetrical cells up to 150 and 200 h at 2.0 and 1.0 mA cm^–2^, respectively. The nanodiamond co-deposition can significantly alter the lithium plating behavior, affording a promising route to suppress lithium dendrite growth in lithium metal-based batteries.

## Abstract (web summary)

Lithium metal is an ideal anode material for rechargeable batteries but suffer from the growth of lithium dendrites and low Coulombic efficiency. Here the authors show that nanodiamonds serve as an electrolyte additive to co-deposit with lithium metal and suppress the formation of dendrites.

## Introduction

Lithium (Li), the lightest metal, delivers a theoretical specific capacity of 3860 mAh g^−1^, nearly ten times higher than the traditional graphite anode (372 mAh g^−1^) in Li ion batteries (LIBs). The Li^+^/Li redox couple provides the most negative potential of −3.04 V (vs. standard hydrogen electrode), rendering a high working voltage in a full cell. These features deliver a high-energy density when the Li metal anode is paired with the high-capacity cathode material to form a full cell. As a result, rechargeable Li metal-based batteries (LMBs), such as Li-sulfur (Li-S) and Li-oxygen (Li-O_2_) batteries are regarded as promising candidates for high-energy-density storage^1, 2^. However, LMBs may develop dangerous Li dendrites, limiting their practical applications due to the following reasons^3–7^: (1) dendritic deposition of Li can electronically connect the cathode and anode, resulting in the cell short circuiting, thermal runaway, and failure with possible explosion or fire; (2) Li dendrites increase the contact area, facilitating side reactions between the Li metal and organic electrolyte. The reaction products electronically isolate the Li metal from the conductive matrix, thus resulting in inactive (dead) Li, and, consequently, low Coulombic efficiency, large polarization, and poor lifespan of the LMB^8–10^.

Strategies to suppress Li dendrites can be divided into four categories: (1) solid/gel polymer electrolyte^11–13^, (2) Li metal/organic electrolyte interface modifications^14–20^, (3) weakening space charge on the anode surface^8, 21, 22^, and (4) anode matrix design^23–26^. While extensive studies have been conducted to explore methods to suppress Li dendrite growth, investigations into the mechanism of nucleation and growth of Li metal are limited^27^. Recently, Cui and co-workers investigated the nucleation potential of Li on various current collectors. Their results indicated a substrate-dependent nucleation behavior, as they achieved selective deposition of Li metal onto a chosen substrate^28^. By designing a nanocapsule structure of hollow carbon spheres with nanoparticle seeds inside, they enabled Li metal to plate the inside of the hollow carbon spheres. However, this strategy simply conceals Li deposits (or dendrites) inside a carbon sphere, and does not fully solve the dendrite problem.

It should be noted that dendrite growth is not unique to the field of rechargeable metal batteries. In the conventional electroplating industry, numerous efforts have also been devoted to suppressing the dendritic growth and achieving the uniform deposition of metal coatings, such as Ni and Co^29, 30^. A nanodiamond-involved co-deposition technique by adding nanodiamond particles into the electroplating bath, has been well-developed and applied in industrial electroplating to achieve the deposition of uniform metal films^31, 32^. This technique involves the co-deposition of metal ions and nanodiamond particles, and the underlying mechanism has been thoroughly investigated^33^: (1) The metal ions adsorb on the surface of nanodiamond and are carried to the electrode surface by convection of electrolyte in the electrolytic bath and electric field; (2) Metal ions accept electrons and are reduced to metal deposits on the electrode surface. The adsorbed nanodiamond particles are either released into the solution or captured by the growing metal film. Co-deposition using nanodiamond as an additive leads to the uniform deposition of metal films, and improves the hardness, lubricity, and wear resistance of the deposited film^34, 35^. Hence, a major improvement in properties can be achieved with minimal capture of nanodiamond particles, simply due to modification of the deposition conditions with nanoparticles at the solid-electrolyte interface^36^.

**Fig. 1:**
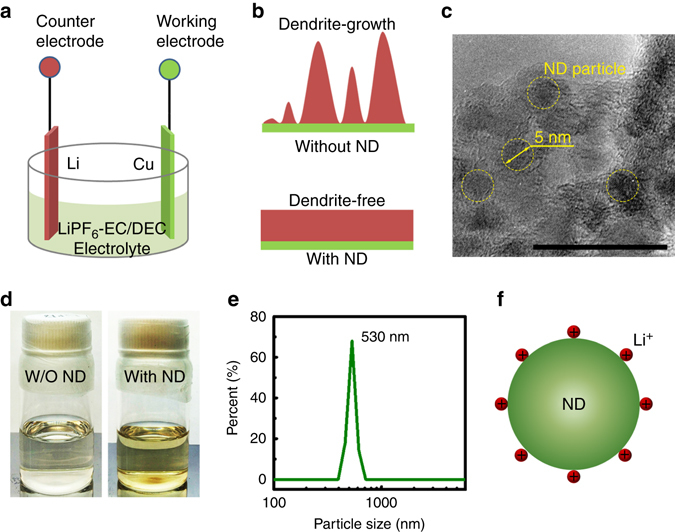
Property of nanodiamond electrolyte and its application in Li ion electroplating. Schematic illustration showing **a** the electroplating bath and **b** the role of nanodiamond additives in suppressing Li dendrite growth. **c** TEM image of nanodiamond particles. The *scale bar* is 20 nm. **d** Optical images of lithium hexafluorophosphate (LiPF_6_)-ethylene carbonate (EC)/diethyl carbonate (DEC) electrolyte without and with nanodiamond additives (0.82 mg mL^−1^). **e** Size intensity distribution of nanodiamond agglomerates in LiPF_6_-EC/DEC electrolyte. **f** Schematic illustration of nanodiamond particles with adsorbed Li ions from the electrolyte. The word ‘‘ND’’ in the figure is the abbreviation of ‘‘nanodiamond’’

Inspired by this co-deposition strategy, we propose the use of nanodiamond additives in a conventional LIB electrolyte, lithium hexafluorophosphate (LiPF_6_)-ethylene carbonate (EC)/diethyl carbonate (DEC) electrolyte, to suppress Li dendrite growth. Similar to a typical electroplating cell, a two-electrode system, with a Cu foil as the cathode and a Li foil as the anode, is designed to probe Li metal deposition behavior. Parts of Cu and Li foils are immersed in the electrolyte (Fig. 1a). The nanodiamond particles, modified by octadecylamine (ODA) groups, are added to and dispersed in the ester-based electrolyte. After adding nanodiamonds to the electrolyte, Li ions co-deposit with nanodiamond particles onto the substrate, producing uniform and dendrite-free Li deposits, and, therefore, resulting in stable electrochemical cycling (Fig. 1b).

## Results

### Nanodiamond electrolyte

Nanodiamond particles used here were produced by a commercial detonation method at a low cost, then carboxylated and subsequently modified by covalent linking of ODA^37, 38^. They have a crystal size of ~5 nm and high crystallinity (Fig. 1c and Supplementary Fig. 1). The interplanar crystal spacing in lattice-fringe transmission electron microscopy (TEM) images was measured to be ~ 0.21 nm, which corresponds to diamond (111) planes (0.206 nm, PDF#65-0537). An EC/DEC electrolyte with dispersed modified nanodiamond particles and a saturation concentration of 0.82 mg mL^−1^ was prepared. Compared to the original colorless and transparent EC/DEC electrolyte, the solution became light-yellow after nanodiamond particle addition (Fig. 1d). Aggregation of nanodiamond particles cannot be fully avoided in the electrolyte and nanodiamond clusters with a size of ~530 nm were measured by dynamic light scattering (Fig. 1e). This size of nanodiamond clusters was largely reduced compared to the pristine commercial nanodiamond^39^. The solution color and size distribution of nanodiamond clusters in the electrolyte did not change after 2 months, indicating the stability of the nanodiamond dispersion in the electrolyte (Supplementary Fig. 2). The nanodiamond particles are able to adsorb Li ions onto their surfaces (Fig. 1f) and co-deposit onto the Cu foil with Li metal, thus acting as the nucleation seeds that guide Li ion deposition. Furthermore, the charged nanodiamond particles do not aggregate inside the electrochemical cells during the practical charging/discharging processes.

### Li plating morphology

**Fig. 2:**
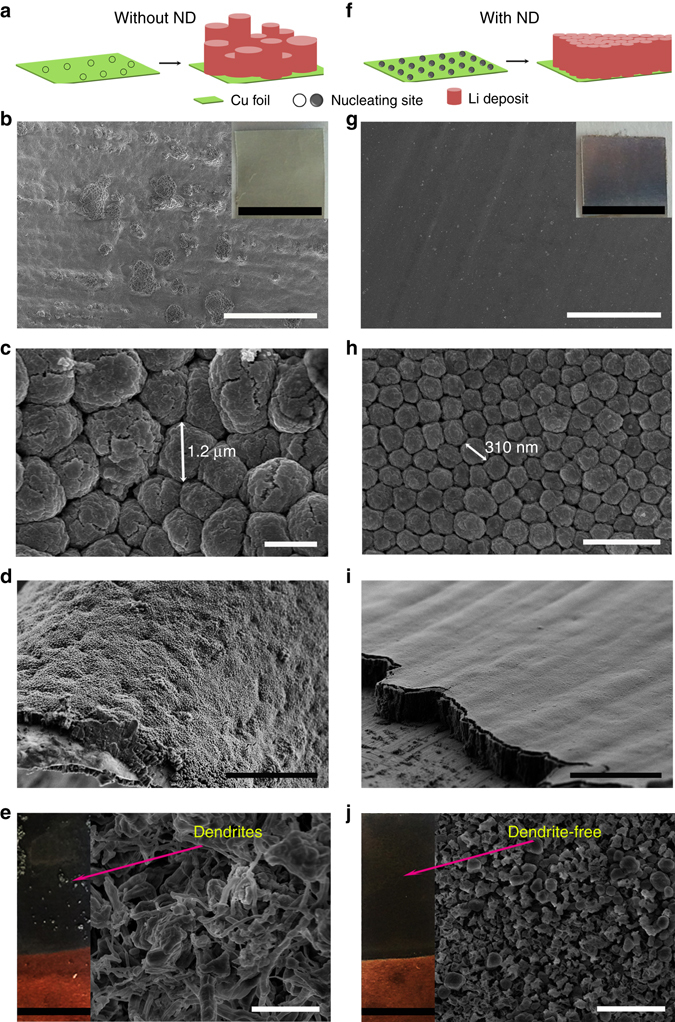
Morphology of Li deposits after galvanostatic plating. Schematic illustration describing Li ion plating behavior in the LiPF_6_-EC/DEC electrolyte **a** without and **f** with nanodiamond additives. SEM images of **b**–**e** Li deposits in LiPF_6_-EC/DEC electrolyte without nanodiamond additive. SEM images of **g**–**j** Li deposits in LiPF_6_-EC/DEC electrolyte with nanodiamond additives. **b**–**d** and **g**–**i** Li plating after one time at 0.5 mA cm^−2^ and with plating time of 6 h. **e**, **j** Li plating after three cycles at 0.5 mA cm^−2^ and with each step time of 6 h. The *insets* in **e**, **j** are the optical images of the corresponding Li deposits. The *scale bars* in **b** and **g**, **c** and **h**, **d** and **i**, **e** and **j** are 100, 1, 50, 5 μm, respectively. The *scale bars* in the *insets* of **b**, **g**, **e**, and **i** are 1 μm. The word ‘‘ND’’ in the figure is the abbreviation of ‘‘nanodiamond’’

Figure 2 illustrates the role of nanodiamond additives in Li ion deposition behavior. Stripping of Li ions from the Li foil to plate on the Cu foil was conducted in an electrolytic bath with or without nanodiamond additives (Fig. 1a). The Cu foil surface before plating was clean and even (Supplementary Fig. 3). The morphologies of Li deposits after the first Li ion plating process (discharging at 0.5 mA cm^−2^ for 6 h) were shown in Fig. 2a–d and f–i. Without nanodiamond in the electrolyte, an uneven morphology is clearly shown, as many bumps were observed on the surface of deposited Li films (Fig. 2b–d). After introducing nanodiamond into the electrolyte, uniform deposition of Li metal on Cu foil was achieved, as indicated by a bright metallic luster caused by the uniform and dense metal surface (Fig. 2g–i). Since no 500-nm diamond cluster could be observed on the Li surface or incorporated into Li, we assume that only a small number of individual nanometer-sized nanodiamond particles were captured by the growing Li film, if any at all.

The Li deposits appear to have a columnar structure (Supplementary Fig. 4). In the electrolyte without nanodiamond, the deposited Li columns had an average diameter of 0.7 ~ 0.8 μm (Fig. 2c, Supplementary Fig. 5a, b). Li columns with a small average diameter (0.3 ~ 0.4 μm) were obtained in the nanodiamond-containing electrolyte (Fig. 2h, Supplementary Fig. 5e, f), leading to a dendrite-free morphology^40^. The colloidal solution remains stable after the nanodiamond electrolyte was stored for 2 months, and can still keep the uniform columnar structure (Supplementary Fig. 6). It is important to mention that the size of those columns is smaller than the size of nanodiamond aggregates in solution, again suggesting that these 500-nm nanodiamond aggregates break apart during Li plating. The difference in the crystal size of Li deposits was analyzed by X-ray diffraction (XRD) (Supplementary Fig. 7). A wider peak of Li (110) of Li deposits in the nanodiamond electrolyte confirms their smaller crystal size, compared to that in the nanodiamond-free electrolyte (Supplementary Note 1). The reduced size of Li deposits in the nanodiamond electrolyte is ascribed to the increased number of nucleation sites that are induced by the nanodiamond particles^33^. The arrayed morphology of Li deposits can be well maintained when the current density is increased to 1.0 mA cm^−2^ for 3.0 h (3.0 mAh cm^−2^) (Supplementary Fig. 8).

After the third Li plating and stripping (charging and discharging at 0.5 mA cm^−2^ for three cycles with each step time of 6 h), many optically visible particles on Li deposits were observed in the nanodiamond-free electrolyte (Fig. 2e). Imaging via high-magnification scanning electron microscopy (SEM) revealed that these particles were dendritic Li clusters (Fig. 2e). In comparison, the dendrite-free morphology of Li deposits was observed in the nanodiamond-containing electrolyte (Fig. 2j). These results clearly indicate that nanodiamond additives successfully induce smaller crystal sizes of Li deposits, leading to a smooth surface and dendrite-free morphology.

Li morphologies were studied after 1^st^, 2^nd^, 5^th^, 10^th^, 20^th^, and 50^th^ cycles at 0.5 mA cm^−2^ with plating/stripping time of 1 h in each cycle (Supplementary Fig. 9). Although some coarsening appears after 20 and 50 cycles, the primary morphology was maintained after 100 h Li plating/stripping at a relatively high-current density (0.5 mA cm^−2^). This finding indicates that nanodiamond particles both remain available in the electrolyte and are able to preserve dendrite-free morphology, even after long-term cycling.

X-ray photoelectron spectroscopy (XPS) of Li deposits after Li plating/stripping confirmed that superior long-term stability is induced by the recyclability of nanodiamond during Li plating and stripping (Supplementary Fig. 10). Relative to nanodiamond-free electrolyte, Li in nanodiamond-containing electrolyte displays a new peak, which originates from the co-deposited nanodiamond particles. The co-deposition of nanodiamond particles and Li was also confirmed by the carbon enrichment in the deposited Li layer (Supplementary Fig. 11, Supplementary Note 2 **)**. After Li stripping, the nanodiamond peak in the XPS spectrum disappears, demonstrating the recyclability of nanodiamond during Li plating and stripping. Additionally supporting their recyclability, nanodiamond particles can not only co-deposit with Li ions, but also strip off from the Cu substrate to render the long-term stability of Li plating morphology.

Diluted (0.41 mg mL^−1^) and concentrated (4.1 mg mL^−1^) nanodiamond electrolytes were prepared to investigate the effect of nanodiamond concentration on the Li plating morphology (Supplementary Fig. 12). In the 0.41 mg mL^−1^ nanodiamond electrolyte, Li deposits were less uniform, containing clearly smooth regions and regions with a few bumps (the size of Li deposits is *ca*. 2.6 μm) (Supplementary Fig. 13). This can be ascribed to an insufficient number of nucleation sites. Even in the smooth regions, the size of Li crystals varied considerably, ranging from several hundred nanometers to a few microns with an average size of 0.6 ~ 0.7 μm (Supplementary Figs 5c, d and 13c). An interesting phenomenon is that the size of Li in bumpy regions was always larger than that in the smooth regions. This emphasizes the importance of reducing the size of Li crystals by providing a large number of nucleation sites. In the 4.1 mg mL^−1^ nanodiamond electrolyte, Li deposits had an average size of 0.9 ~ 1.2 μm (Supplementary Fig. 5g, h and 14), which is more uniform than those in the 0.41 mg mL^−1^ nanodiamond electrolyte. However, the smoothness in the 4.1 mg mL^−1^ nanodiamond electrolyte was worse than that in the 0.82 mg mL^−1^ nanodiamond electrolyte. This is because the dispersion contains a large amount of nanodiamond aggregates, and is consequently oversaturated and unstable. The observed concentration dependence highlights the importance of a high concentration and a uniform dispersion of nanodiamond particles in the electrolyte, which may be improved by modifying the functionalization of nanodiamond and controlling the size of the aggregates.

### Interaction between Li ions and nanodiamond

**Fig. 3:**
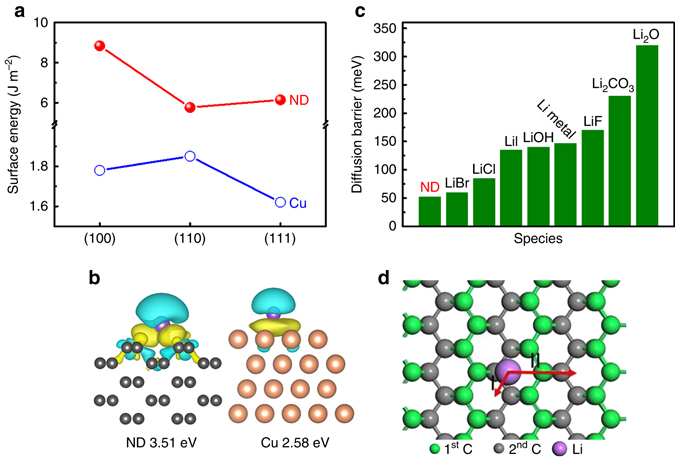
First-principle calculations to describe Li ion plating behavior on nanodiamond surface. **a** Surface energies of low index facets for nanodiamond and Cu. **b** Differences of charge density for Li on nanodiamond (110) and Cu (111) surfaces. The *turquoise* and *yellow* regions indicate depletion and accumulation of electrons, respectively. **c** Diffusion barrier of Li on different surfaces. Except for nanodiamond, the diffusion barriers of other materials are cited from the ref. ^41^. **d** The most stable adsorption sites and diffusion paths for Li on nanodiamond (110) surface. The word ‘‘ND’’ in the figure is the abbreviation of ‘‘nanodiamond’’

To better understand the nanodiamond-guided Li plating behavior, first-principle calculations were performed. We first calculated the surface energies of several low index facets for nanodiamond and Cu to find the most stable and dominating surfaces (Fig. 3a). The results indicate that nanodiamond (110) and Cu (111) are the dominating surfaces for each crystal with the lowest surface energies of 5.76 and 1.62 J m^−2^, respectively. Therefore, nanodiamond (110) and Cu (111) were chosen as the base surfaces for the following discussions of binding energy and diffusion energy barriers.

The binding energies of Li on nanodiamond (110) and Cu (111) surfaces were calculated to be 3.51 and 2.58 eV, respectively (Fig. 3b). The large charge transfer between Li and nanodiamond (110) surface contributes to its high-binding energy. The nearly 1 eV higher binding energy for nanodiamond and Li ions results in a stronger preferential adsorption of Li ions on the nanodiamond surface rather than onto the Cu surface during Li plating. After adsorption, Li ions can either aggregate into a large dendrite, or distribute uniformly and form dendrite-free Li deposits. To investigate Li ion diffusion behavior, the diffusion barrier of Li on nanodiamond was calculated and compared with that on other materials published in literature (Fig. 3c, d)^41^. Compared with these materials, nanodiamond has the lowest Li diffusion energy barrier. This indicates that at the interface of the cathode (Cu foil) and electrolyte, Li ions are inclined to adsorb onto the nanodiamond surface and weaken aggregation and can easily diffuse and distribute uniformly to produce a dendrite-free morphology^42, 43^.

### Electrochemical cycling performance

**Fig. 4:**
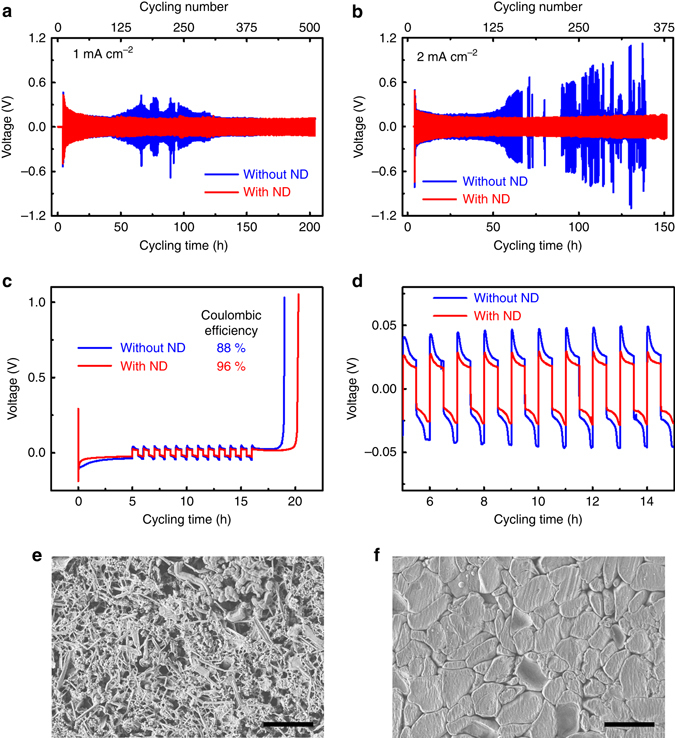
Long-term electrochemical cycling stability. Charge-discharge curves of symmetrical Li | Li cells at **a** 1 mA cm^−2^ and **b** 2 mA cm^−2^. Each charge and discharge time is set as 12 min. **c** Voltage-time curves to calculate the average Coulombic efficiency of Li | Cu cells at 0.5 mA cm^−2^. **d** The enlarged view of **c** from 5 ~ 15 h. The morphology of a Li deposit cycled at 0.5 mA cm^−2^ in the electrolyte **e** without and **f** with the nanodiamond additive. The *scale bars* in **e** and **f** are 10 μm. The word ‘‘ND’’ in the figure is the abbreviation of ‘‘nanodiamond’’

The long-term electrochemical cycling stability of Li electrodes was explored by testing symmetrical Li | Li cells. As shown in Fig. 4a, b, symmetrical Li | Li electrodes have stable cycling in the nanodiamond electrolyte for 200 and 150 h (tests were stopped at that point) at 1.0 and 2.0 mA cm^−2^, respectively, exhibiting stable Li metal deposition, though with a little increase in the polarization (100 mV at 1 mA cm^−2^ to 120 mV at 2 mA cm^−2^). In comparison, symmetrical Li | Li electrodes in the nanodiamond-free electrolyte have obvious fluctuations in voltages caused by the ever-changing and increasing interfaces of the Li metal and electrolyte. At a high-current rate (2.0 mA cm^−2^), the polarization of the nanodiamond-free electrolyte is much larger than that of the nanodiamond electrolyte, due to Li dendrite growth and dead Li. Additionally, electrochemical impedance (EIS) spectroscopy for the Li | Li cells at 1.0 mA cm^−2^ was conducted after 10, 30, 40, 50, 60, 70, and 80 cycles (Supplementary Fig. 15). After ten cycles, cells in the nanodiamond electrolyte exhibited a stable Li ion diffusion resistance of ~ 84 Ω, while the impedances of nanodiamond-free electrolytes fluctuated largely, ranging between 219 and 97 Ω, thus demonstrating a stable interfacial impedance induced by the nanodiamond-containing electrolyte.

When decreasing the nanodiamond concentration from 0.82 to 0.41 mg mL^−1^, the cells also retained good stability after voltage was applied (Supplementary Fig. 16a). The 0.41 mg mL^−1^ nanodiamond electrolyte showed much better performance than the nanodiamond-free electrolyte. While the 0.82 mg mL^−1^ electrolyte showed similar cycling stability, it exhibited less increase in the voltage polarization with extended cycles. These results clearly demonstrate the role of nanodiamond in stabilizing Li metal to achieve a stable long-term cycling performance.

The Coulombic efficiency during Li plating/stripping was probed in a Li | Cu cell according to Aurbach et al.^44^ (Fig. 4c). During 12 cycles, the average Coulombic efficiency of cells in the nanodiamond-containing electrolyte (0.82 mg mL^−1^) was 96%, which is much higher than that in the nanodiamond-free electrolyte (88%). The higher Coulombic efficiency in the nanodiamond-containing electrolyte indicates a higher Li utilization during Li plating/stripping. When the cycling time and number were extended to 100 cycles (200 h), a stable performance was maintained with an average Coulombic efficiency of 96% (Supplementary Fig. 17). For the reduced nanodiamond concentration of 0.41 mg mL^−1^, an average Coulombic efficiency of 95% was achieved (Supplementary Fig. 16b), which is only slightly smaller than the 0.82 mg mL^−1^ nanodiamond electrolyte (96%), but still much higher than that of the nanodiamond-free electrolyte (88%). While close to 100% efficiency is expected in commercial batteries, we assume that a lower efficiency in our experiment does not result from the electrolyte breakdown. It rather comes from some Li staying adsorbed on nanodiamond particles due to the strong binding energy between nanodiamond and Li. Still, the nanodiamond additive also renders a reduced polarization of 19 mV during Li plating/stripping, while it is 29 mV for nanodiamond-free electrolyte (Fig. 4d). In the nanodiamond-containing electrolyte, the electrode starts to plate Li at ~ −15 mV, and strips Li at ~ 27 mV. However, in the nanodiamond-free electrolyte, the plating and stripping processes start at approximately −21 and 48 mV, respectively. Thus, nanodiamond particles in the electrolyte effectively promote Li nucleation and dissolution.

SEM imaging of cycled electrodes revealed a large density of dendrites in the nanodiamond-free electrolyte (Fig. 4e), especially compared to the electrode in the nanodiamond-containing electrolyte. These dendrites lead to an unstable Li metal/electrolyte interface, the formation of dead Li, and poor long-term cycling performance. In the nanodiamond-containing electrolyte, the electrode shows a dendrite-free morphology (Fig. 4f). There are two key differences between the Li plating morphologies in the electroplating bath and the coin cells. Firstly, the plating morphology in the coin cells was flattened due to pressure, demonstrating the importance of the electroplating bath in investigating the original Li plating morphology. Secondly, the Li crystal size in the coin cells after many cycles grew larger than that in the electroplating bath. The increased crystal size can be attributed to the aggregation of nanodiamond particles during long-term cycling. Therefore, it is important to produce well dispersed and aggregate-free nanodiamond particles in the electrolyte.

Li | LiFePO_4_ (LFP) full cells were assembled to test the viability of nanodiamond-containing electrolyte in the practical batteries (Supplementary Fig. 18). After activation at 0.1 C (1.0 C = 180 mA g^−1^), testing of the LFP battery with nanodiamond electrolyte indicated a very stable cycling at 1.0 C with a capacity decay of 4.9% after 130 cycles, while the cell with nanodiamond-free electrolyte exhibited a larger capacity decay of 14.2% (Supplementary Fig. 18a). As these cells have the same cathode, the capacity decay can be attributed to the anode depletion. The superior cycling stability of the LFP battery with nanodiamond-containing electrolyte is ascribed to dendrite-free Li deposits and a stable electrode-electrolyte interface on the Li metal anode.

The morphologies of the LFP cathode, Celgard 2400 separator, and Li foils were investigated after 5 and 20 cycles. The LFP morphologies were remarkably similar, with conductive agents (acetylene black) connecting LFP particles (Supplementary Fig. 19). Relative to the pristine separator, the separators after tests maintained their porous structure (Supplementary Fig. 20). There were no obvious nanodiamond particles on the LFP and separator surface, demonstrating that nanodiamond particles were not be absorbed in noticeable accounts onto the high-surface-area LFP cathode and the separator, hence keeping the stable nanodiamond concentration in the electrolyte. The cycled Li morphologies after 5 and 20 cycles (Supplementary Fig. 21) were similar to that in the Li | Cu coin-cell (Fig. 4f). After many cycles, the nanodiamond electrolyte was still able to maintain the arrayed and dendrite-free Li deposits in the Li | LFP cells. Therefore, nanodiamond particles can effectively maintain a stable Li-electrolyte interface and dendrite-free Li morphology in commercial electrolytes, while having little adverse effects on the cathode and separators.

The role of nanodiamond concentration on the full-cell cycling performance was also investigated (Supplementary Fig. 18b). Similar to Li | Li and Li | Cu cells, the Li | LFP cells of both 0.41 and 0.82 mg mL^−1^ nanodiamond electrolyte indicated a good cycling stability (capacity decay rate: 4.4%), but the cycling capacity of the 0.41 mg mL^−1^ nanodiamond-electrolyte was 10 mAh g^−1^ lower than that of the 0.82 mg mL^−1^ nanodiamond electrolyte. These results indicate that both nanodiamond concentrations can lead to dendrite-free Li deposits and a stable Li-electrolyte interface, but the smaller nanodiamond concentration will not provide sufficient number of nucleation sites for uniform Li plating.

## Discussion

**Fig. 5:**
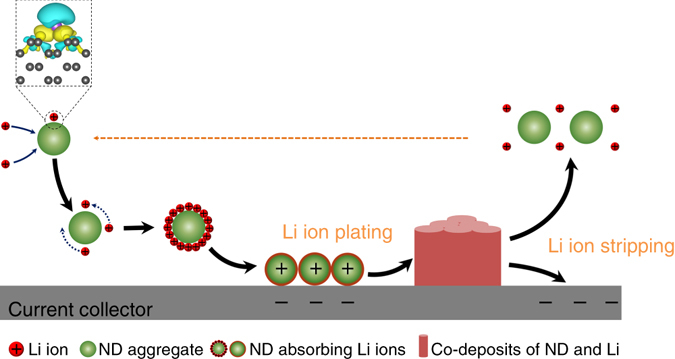
Schematic illustrating the co-deposition of Li ions on nanodiamond, growth of the columnar Li film and the stripping of Li deposits. The word ‘‘ND’’ in the figure is the abbreviation of ‘‘nanodiamond’’

Nanodiamond shows a potential to suppress Li dendrites growth by acting as heterogeneous seeds for Li plating. Its critical role in Li ion plating can be described in four steps: (Fig. 5) (1) Li ions adsorb on nanodiamond, rather than Cu, due to the difference in binding energy and a large surface area of nanodiamond; (2) Nanodiamond particles with Li ions are transported to the surface of the Cu foil under solution-forced convection and electric field force; (3) As a heterogeneous seed, nanodiamond renders initial Li nucleation; (4) Due to the small sizes of nanodiamond particles, nanodiamond-guided Li deposits have small crystal sizes and uniform morphology. After that, the co-deposits of nanodiamond particles and Li ions can successfully strip off to the electrolyte during the Li stripping processes to maintain a stable concentration of nanodiamond in the electrolyte and provide long-term cycling stability of the Li metal anode.

Diamond nanoparticles co-deposition is an existing technology in the metal electroplating industry. Therefore, we propose that this commercially viable method can be transferred to the battery industry. Dielectric diamond nanoparticles do not represent a threat; even if they penetrate through the separator, they cannot short-circuit the device or increase leakage current. However, several issues must be addressed before their practical application in LMBs. (1) Compared to the high concentration in the aqueous solution (10 ~ 20 mg mL^−1^) used in the electroplating industry, the saturation concentration of nanodiamond in the LiPF_6_-EC/DEC electrolyte is only 0.82 mg mL^−1^, though we used an ODA-modified hydrophobic nanodiamond. More efforts are required to decrease the particle size in solution and improve the solubility of nanodiamond in the electrolyte. This task has been accomplished for aqueous dispersions^38^ and dispersion in organic electrolytes should be possible, too. (2) In the metal electroplating industry, trial-and-error tests have been used to choose the best additive for electrodeposition. Effects of other insulating nanoparticles, such as Al_2_O_3_, SiO_2_, BN, or AlN, on the Li plating behavior should also be evaluated.

Besides their demonstrated potential in co-deposition with Li ions to suppress Li dendrite growth, nanodiamond particles may also be utilized as an electrolyte additive to regulate the solid electrolyte interphase (SEI) film. Similar to LiF^17^, SiO_2_^45^, and Al_2_O_3_^46^ particles in the surface film of Li deposits, nanodiamond particles form a low Li ion diffusion barrier and ensure a fast Li ion diffusion rate. When nanodiamond particles deposit on the anode surface, they can strengthen the pristine SEI film to protect the Li metal anode and render the practical application of the Li metal anode. In this work, a commercial Li ion electrolyte was used as a dispersant without any additional additives. When nanodiamond functions synergetically with other SEI-forming additives, such as fluoroEC^47^, Li polysulfide and LiNO_3_^48^ etc., the Coulombic efficiency and Li depositing morphology could be further enhanced. Here, we only present a feasibility study showing that the electroplated particles can be applied to the Li metal anode. After probing the mechanism and coupling with the SEI-forming additives, electroplated particles are expected to be used in next-generation commercial Li metal batteries.

In summary, in this work, we propose the nanodiamond-assisted suppression of Li dendrites growth in LMBs. During Li plating, nanodiamond particles serve as heterogeneous nucleation seeds and adsorb Li ions. Due to the low diffusion energy barrier of Li ions on the nanodiamond surface, the adsorbed Li ions lead to formation of uniform Li deposits, rather than large Li dendrites, which have smaller crystal sizes than that obtained in the nanodiamond-free electrolyte. The dendrite-free morphology leads to an enhanced electrochemical performance. The nanodiamond-modified electrolyte provides stable cycling of Li | Li cell for 200 h at 1 mA cm^−2^, 150 h at 2 mA cm^−2^ and a high Coulombic efficiency of 96% in Li | Cu cells (88% for nanodiamond-free electrolyte). The nanodiamond-assisted co-deposition strategy presents a promising method for suppressing Li dendrite growth in LMBs.

## Methods

### Nanodiamond modification

Nanodiamond particles were modified following the published method^37^. 1.5 g of nanodiamond (UD90 grade, provided by SP3, USA), was purified by air oxidation at 425 °C and cleansed of metal impurities by boiling in a mixture of HCl, HNO_3_, and distilled water for 24 h. 1.5 g of the resulting material was refluxed with 50 mL of SOCl_2_ (Sigma Aldrich) and 1 mL of anhydrous *N*,*N* dimethylformamide (Sigma Aldrich), which is well known as a catalyst for this reaction, at 70 °C for 24 h. After removing supernatant by distillation, the obtained solid was washed with anhydrous tetrahydrofuran three times and then dried at ambient temperature in a desiccator under vacuum. The chlorinated nanodiamond powder was then stirred in a sealed flask with 5 g of ODA (Sigma Aldrich) at 90 ~ 100 °C for 96 h. After cooling, excess ODA was removed by rinsing 4 ~ 5 times with hot, anhydrous methanol (Sigma Aldrich). The ODA-modified nanodiamond can be easily dispersed in organic solvents^37^.

### Nanodiamond-containing electrolyte preparation

In an Ar-filled glove box, 50 mg of the obtained ODA-functionalized nanodiamond particles were dispersed in 10 mL of 1.0 M lithium hexafluorophosphate (LiPF_6_), which was dissolved in EC and DEC with a volumetric ratio of 1:1. The obtained colloidal solution was then tightly sealed and transferred to an ultrasonic bath for 3 h to achieve a good dispersion of nanodiamond in the LiPF_6_-EC/DEC electrolyte. After ultrasonication, the solution was left in glove box for 1 day to obtain well-dispersed nanodiamond in the supernatant for the electroplating process and electrochemical long-term cycling tests.

### Electroplating process in the bath

The electroplating process of Li onto the Cu foil was conducted galvanostatically in an electroplating bath at a current density of 0.5 mA cm^−2^. The working electrode was a Cu foil (~1 × 7 cm^2^) and the counter electrode was a Li foil (~ 0.8 × 7 cm^2^). After electroplating, the obtained Li deposits were washed in 1,2-dimethoxyethane to remove electrolyte residues. All the experiments were conducted in an Ar-filled glove box with the water and oxygen contents below 0.5 ppm.

### Materials characterization

The morphology of nanodiamond was characterized using a TEM (JEOL JEM-2100, Japan) with an accelerating voltage of 200 kV. The TEM samples were prepared by dispersing powders in ethanol by sonication, depositing several drops of the solution onto a copper grid covered by lacey carbon films, and then drying in air. The particle size distribution of the 0.82 mg mL^−1^ nanodiamond solution in LiPF_6_-EC/DEC electrolyte was measured using a Zetasizer Nano ZS (Malvern Instruments) in 173° scattering geometry. The morphology of Li deposits was characterized using a SEM (Zeiss Supra 50VP, Germany), operated at 3.0 kV. The SEM samples were prepared in the glove box. The XRD patterns of Li deposits were recorded by a powder diffractometer (Rigaku Smart Lab, USA) with Cu Kα radiation at an acquisition rate of 0.2° min^−1^ and 0.5 s dwelling time.

### First-principles calculations

The calculations were based on bare carbon without considering the functional groups on its surface. VASP code^49^ based on density-functional theory was used. The exchange–correlation energy was calculated using the general gradient approximation with the Perdew–Burke–Ernzerhof exchange-correlation functional^50^. The effect of van der Waals interactions was taken into account and implemented in the optimized exchange van der Waals functional B86b of the Becke (optB86b vdW) functional^51^. The plane wave cutoff energy was 400 eV. The convergence condition for the energy was 10^−4^ eV, and the structures were relaxed until the force on each atom was less than 0.01 eV Å^−1^. The ***c*** axis was set as 25 Å to ensure enough vacuum to avoid interactions between two periods. To calculate the diffusion energy barriers of Li on the surfaces of nanodiamond and Cu, we used the climbing image nudged elastic band method (CI-NEB) implemented in VASP^52^. The NEB paths were relaxed until the forces on all atoms were below 0.03 eV Å^−1^.

Surface energies *γ* were defined as:

1 $$\gamma = \left( {{E_{{\rm{slab}}}}-{{N}}{{{E}}_{{\rm{unit}}}}} \right)/2A,$$

where *E*_slab_ is the total energy of the slab, *E*_unit_ is the total energy per unit of nanodiamond or Cu crystal, *N* is the total number of units contained in the slab model and *A* is the area of each surface.

Binding energies *E*_b_ were defined as:

2 $${E_b} = {E_{{\rm{sub + Li}}}} - \left( {{E_{{\rm{sub}}}} + {E_{{\rm{Li}}}}} \right),$$

where *E*_sub+Li_ is the total energy of the substrate with a Li atom, *E*_sub_ is the total energy of the substrate and *E*_Li_ is the total energy of the Li atom.

### Electrochemical cycling tests

To evaluate the electrochemical long-term performance of nanodiamond-containing electrolyte, symmetrical Li | Li cells and Li | Cu cells were assembled in the glove box. These electrodes were tested in the LiPF_6_-EC/DEC electrolyte with and without nanodiamond in standard CR-2032 coin cells. Polypropylene membranes (Celgard 2400) were used as separators. The coin cells were tested in a galvanostatic mode using a battery cycler (Arbin BT-2143- 11U, College Station, TX, USA). The Li | Li cell was operated at 1 and 2 mA cm^−2^ with a plating/stripping time of 12 min. The Li | Cu cell was operated at 0.5 mA cm^−2^. The average Coulombic efficiency was calculated after Aurbach et al.^44^. An initial amount of lithium (2.5 mAh cm^−2^ at 0.5 mA cm^−2^) was deposited on a copper electrode. Then, 10% of this initial amount (0.25 mAh cm^−2^) was stripped and redeposited galvanostatically at 0.5 mA cm^−2^ for 12 cycles. The final stripping process was interrupted when the working electrode potential exceeded 1 V vs. Li/Li^+^. The average cycling efficiency was calculated from the following equation.

3 $$X=\left[ {{Q_{\rm{c}}} - \left( {X{Q_{\rm{l}}} - {Q_{\rm{r}}}} \right)/N} \right]/{Q_{\rm{c}}} \times 100$$

where *X* is the cycling efficiency (%), *N* is the number of cycles, *Q*_c_, *Q*_l_, *Q*_r_ are the charges involved in a single deposition/stripping process (half cycle), initial loading (massive lithium deposition), and final charging (the residual Li), respectively. Li | LiFePO_4_ (LFP) cells were assembled to evaluate the effect of nanodiamond electrolytes in the practical cells. A homogeneous slurry was prepared by mixing LFP, super P, and polyvinylidene fluoride (PVDF) with a mass ratio of LFP: super P: PVDF = 80:10:10, followed by magnetic stirred for 24 h. The slurry was coated onto an Al foil and dried in a vacuum drying oven at 60 °C for 6 h. The as-obtained foil was punched into 13 mm disks as the working electrodes. 1.0 mm thick Li metal foil was employed as the counter electrode. The coin cells were monitored in galvanostatic mode within a voltage range of 2.5 ~ 3.8 V at 1.0 C (1.0 C = 180 mA g^−1^) after one cycle activation at 0.1 C.

### Data availability

The data that support the findings of this study are available from the corresponding author upon request.

## Electronic supplementary material

Supplementary Information
