# Supplementary material for: Nanodiamonds suppress the growth of lithium dendrites
Source: Nat Commun. 2017 Aug 25;8:336. doi: 10.1038/s41467-017-00519-2 (PMC5571184; doi:10.1038/s41467-017-00519-2)
Supplement: Supplementary file 1 — Supplementary Information [file 41467_2017_519_MOESM1_ESM.pdf]

Supplementary Figures

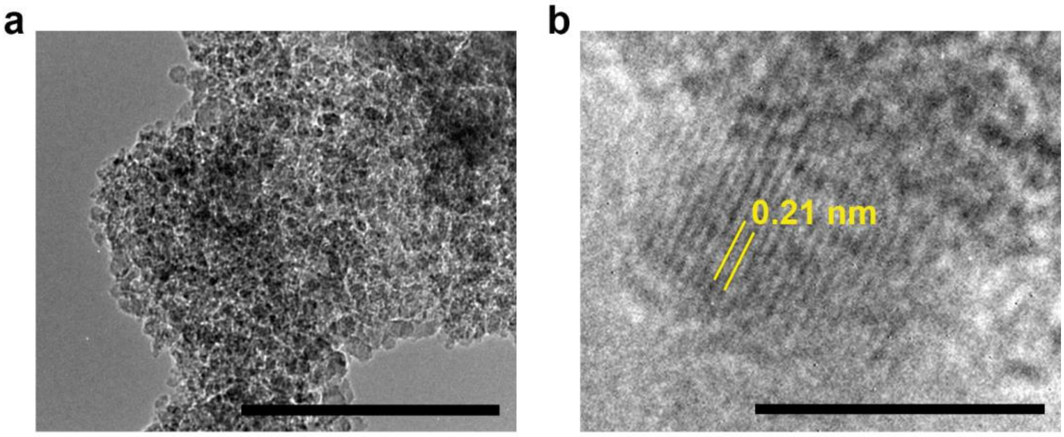

Supplementary Figure 1 | TEM images of nanodiamond particles. The scale bars in (**a**) and (**b**) are 100 and 5 nm.

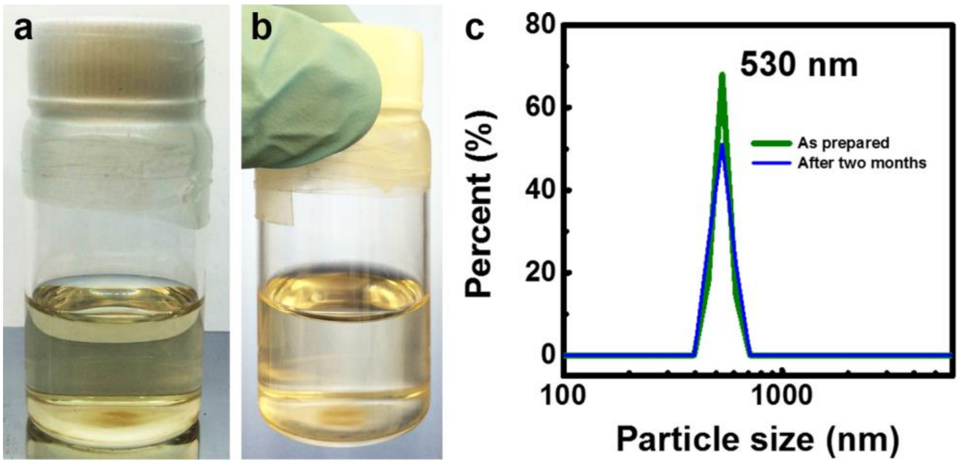

Supplementary Figure 2 | Stability of nanodiamond electrolyte. Optical images of nanodiamond electrolyte (**a**) as prepared and (**b**) after 2 months. (**c**) Size intensity distribution of nanodiamond agglomerates in $LiPF_6$-EC/DEC electrolyte before and after 2 months.

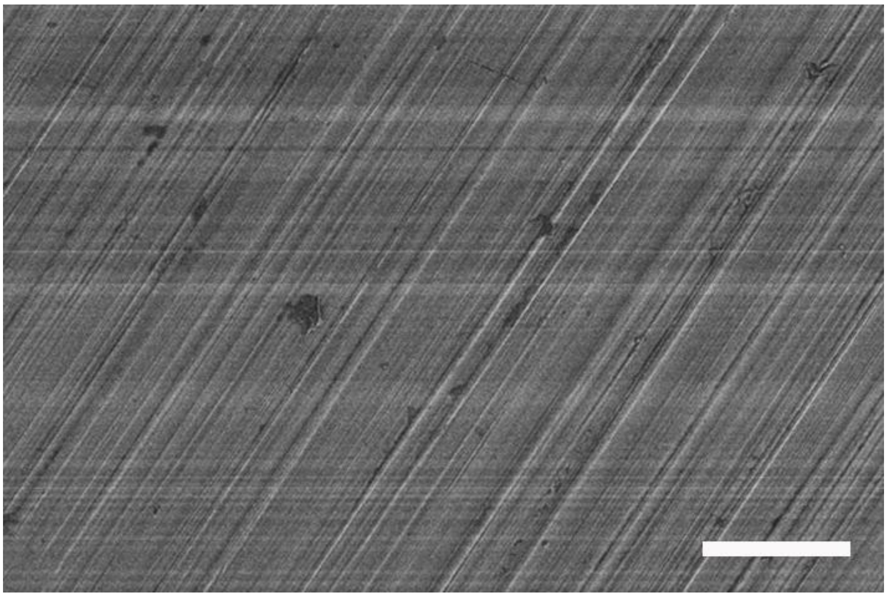

Supplementary Figure 3 | SEM image of Cu foil before Li plating. The scale bar is 50 μm.

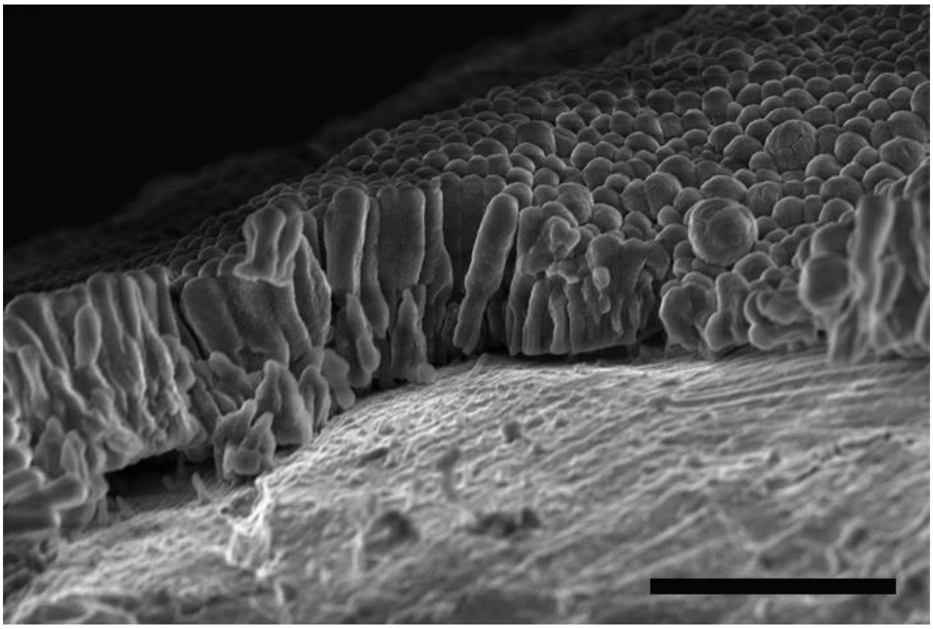

Supplementary Figure 4 | SEM image of first Li deposits with columnar structure in $LiPF_6$-EC/DEC electrolyte. The scale bar is 10 μm.

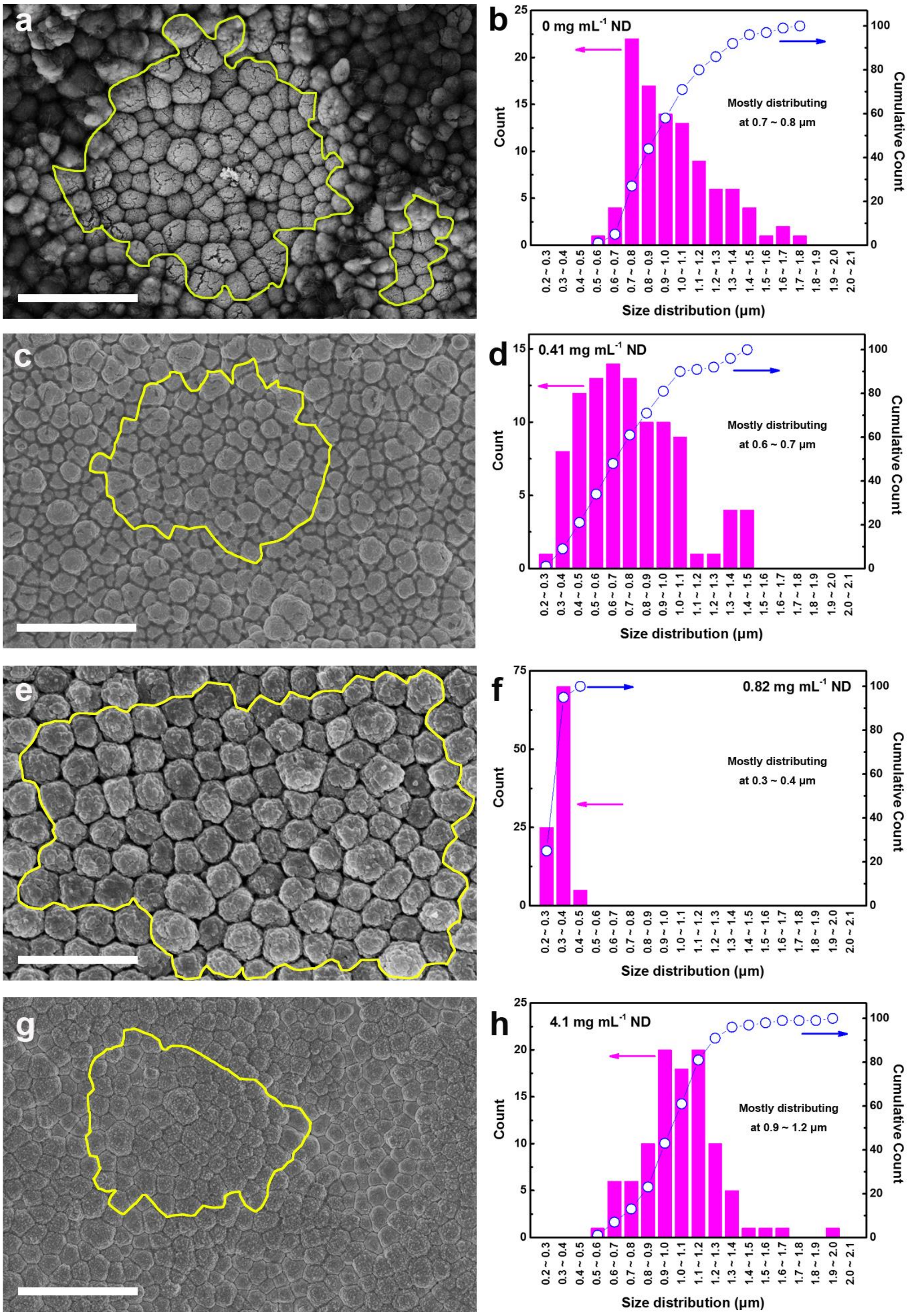

Supplementary Figure 5 | Size distribution of Li deposited in different electrolyte at 0.5 mA cm^{-2} for 6h. Li depositing morphology in (**a**) 0, (**c**) 0.41 mg mL^{-1}, (**e**) 0.82 mg mL^{-1}, (**g**) 4.1 mg mL^{-1} nanodiamond electrolyte, and the corresponding size distributions (**b**) 0, (**d**) 0.41 mg mL^{-1}, (**f**) 0.82 mg mL^{-1}, (**h**) 4.1 mg mL^{-1} nanodiamond electrolyte. The yellow circles in (**a**), (**c**), (**e**), (**g**) are adopted to choose the regions to obtain the size distribution in (**b**), (**d**), (**f**), (**h**). The scale bars in (**a**), (**c**), (**e**), and (**g**) are 5, 5, 1, 5 μm, respectively. The word 'ND' in the figure is the abbreviation of 'nanodiamond'.

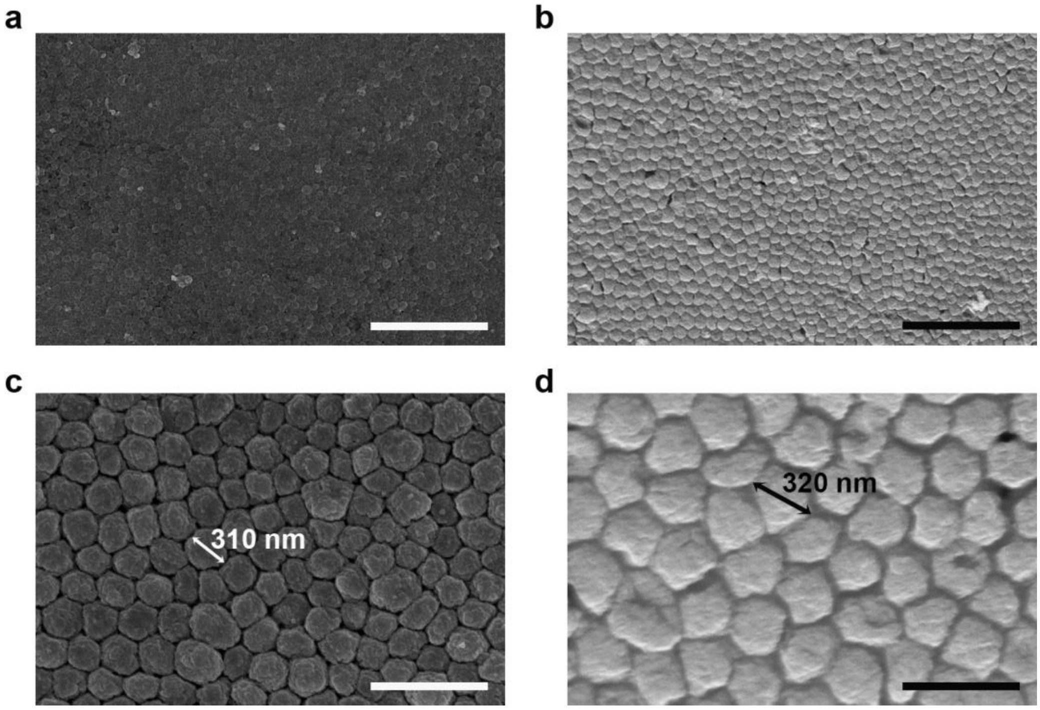

Supplementary Figure 6 | Morphology stability of Li deposits in the same nanodiamond electrolyte. SEM images of Li deposits (**a**) and (**c**) before, (**b**) and (**d**) after 2 months. The scale bars in (**a**) – (**d**) are 5, 2.5, 1, and 0.6 μm, respectively.

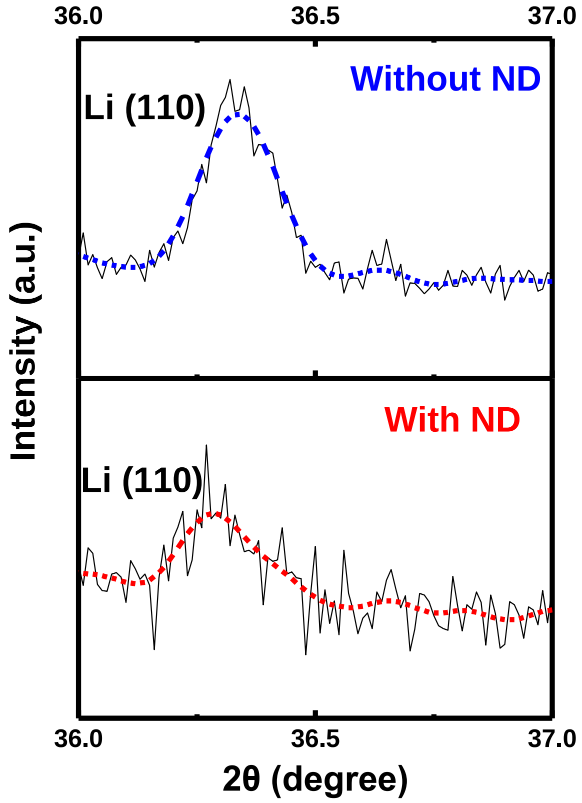

Supplementary Figure 7 | XRD patterns of Li deposits at 0.5 mA cm^{-2}.

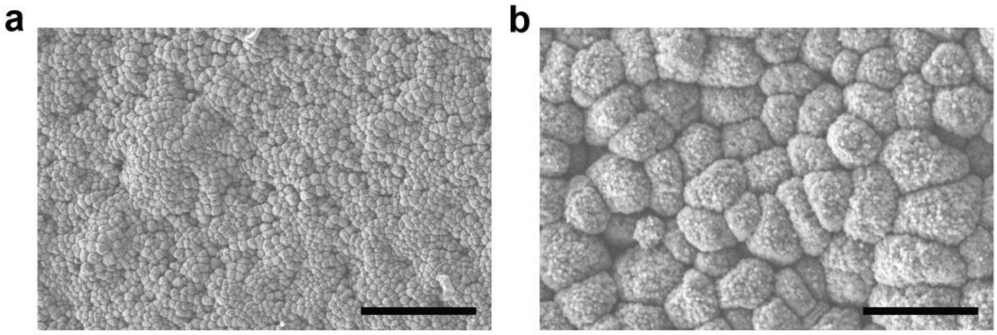

Supplementary Figure 8 | SEM images of Li deposits in nanodiamond-containing electrolyte. (**a**) and (**b**) Li plating morphology at 1 mA cm^{-2} and 3 mAh cm^{-2}. The scale bars in (**a**) and (**b**) are 30 and 5 μm.

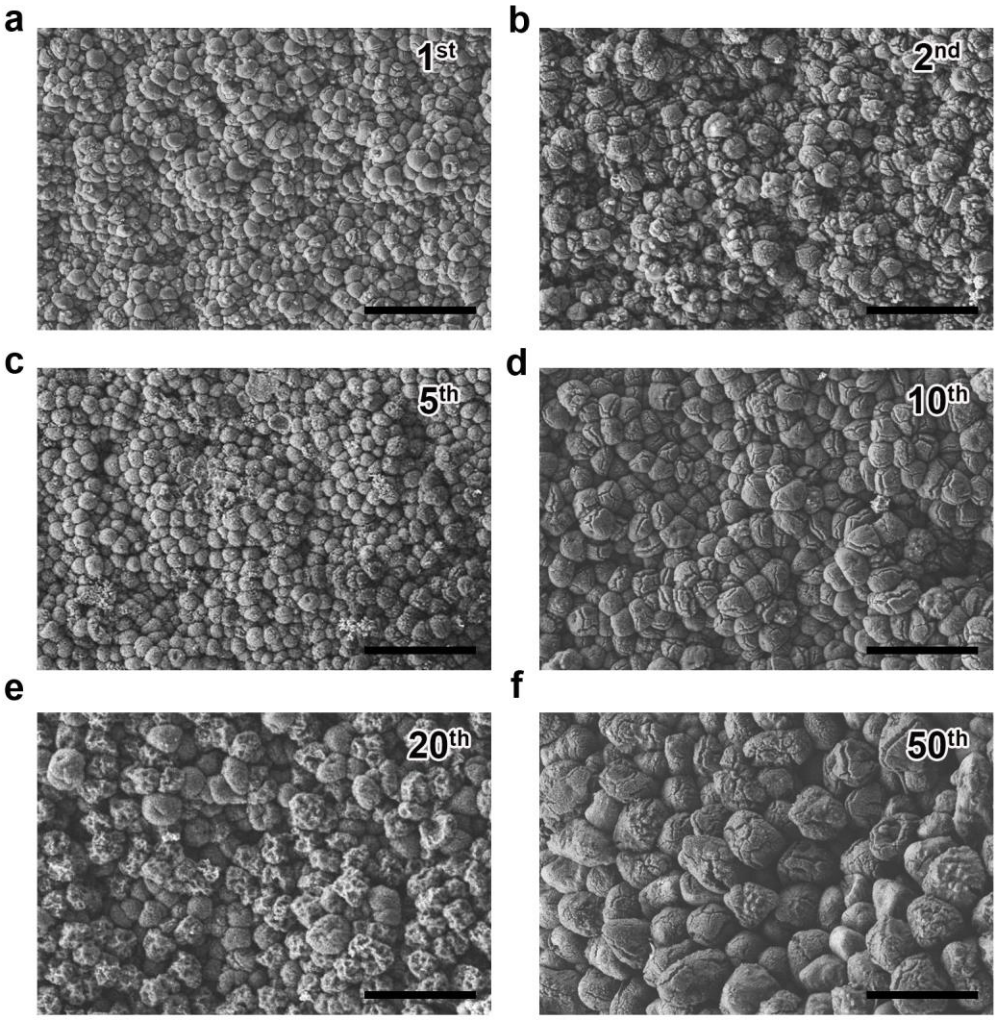

Supplementary Figure 9 | SEM images of Li deposits in nanodiamond-containing electrolyte after many cycles. Li morphology after the (**a**) 1st, (**b**) 2nd, (**c**) 5th, (**d**) 10th, (**e**) 20th, and (**f**) 50th cycle. The scale bars in (**a**) - (**f**) are 10 μm.

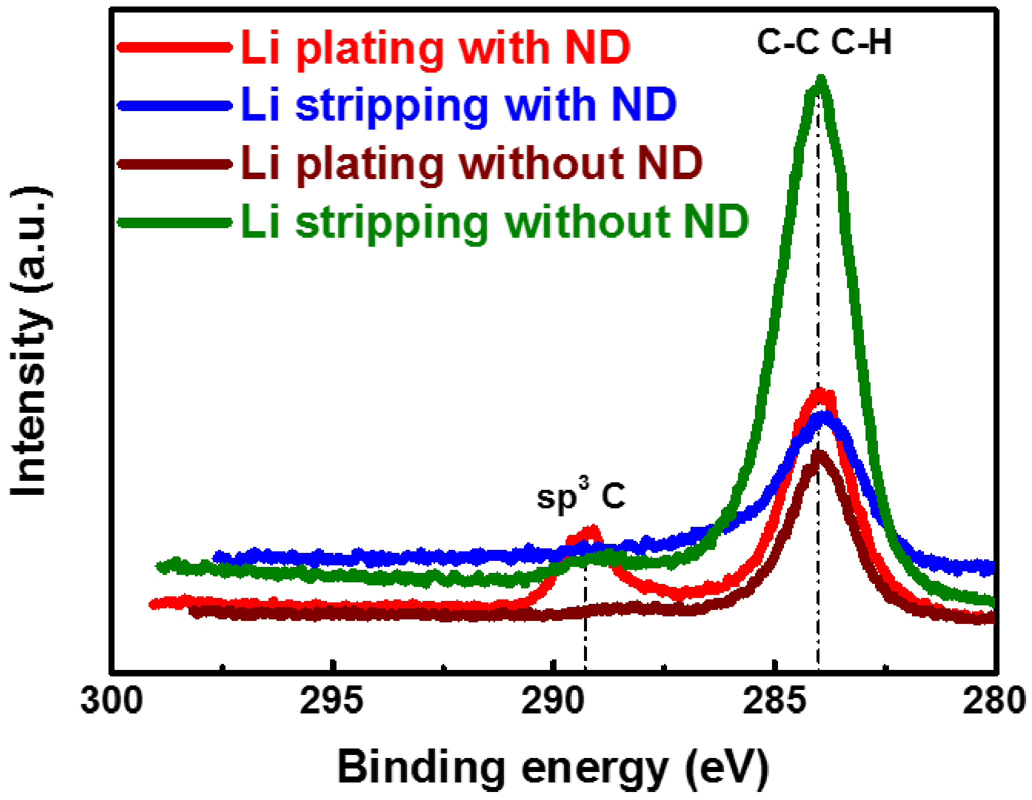

Supplementary Figure 10 | The XPS C1s spectrum of Li anode surface.

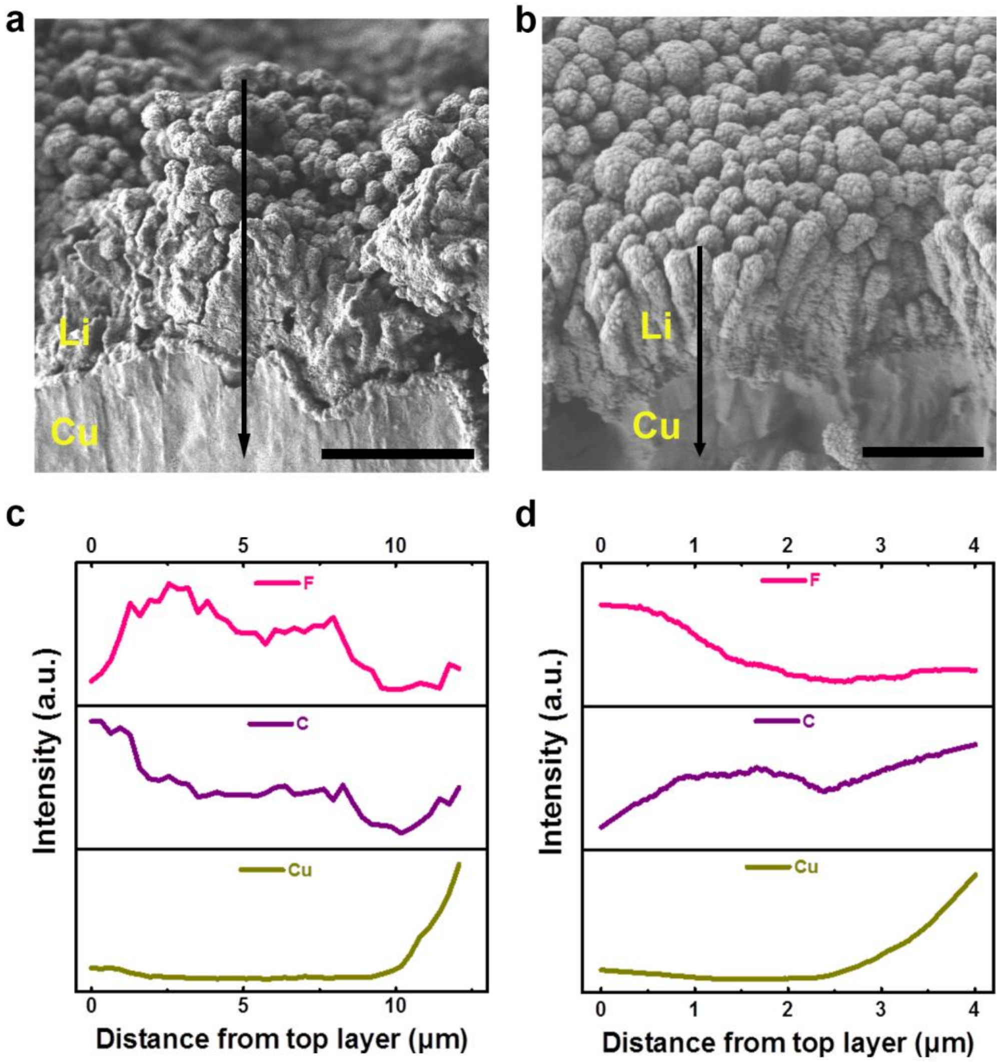

Supplementary Figure 11 | Elemental distribution in the cross-section of Li deposits. The cross-section SEM images of Li deposits (**a**) without nanodiamond and (**b**) with nanodiamond additives, and the corresponding element distributions (**c**) and (**d**). The scale bars in (**a**) and (**b**) are 4 and 2 μm.

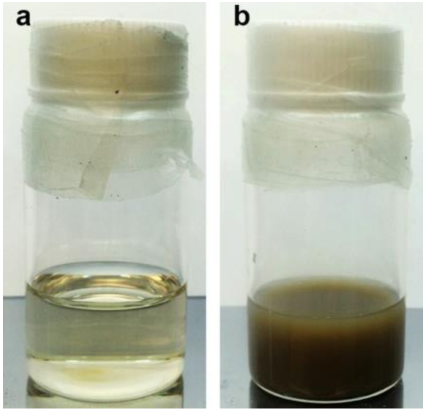

Supplementary Figure 12 | Optical images of $LiPF_6$-EC/DEC electrolyte with different nanodiamond concentrations. (a) 0.41 mg mL^{-1}, (b) 4.1 mg mL^{-1} nanodiamond.

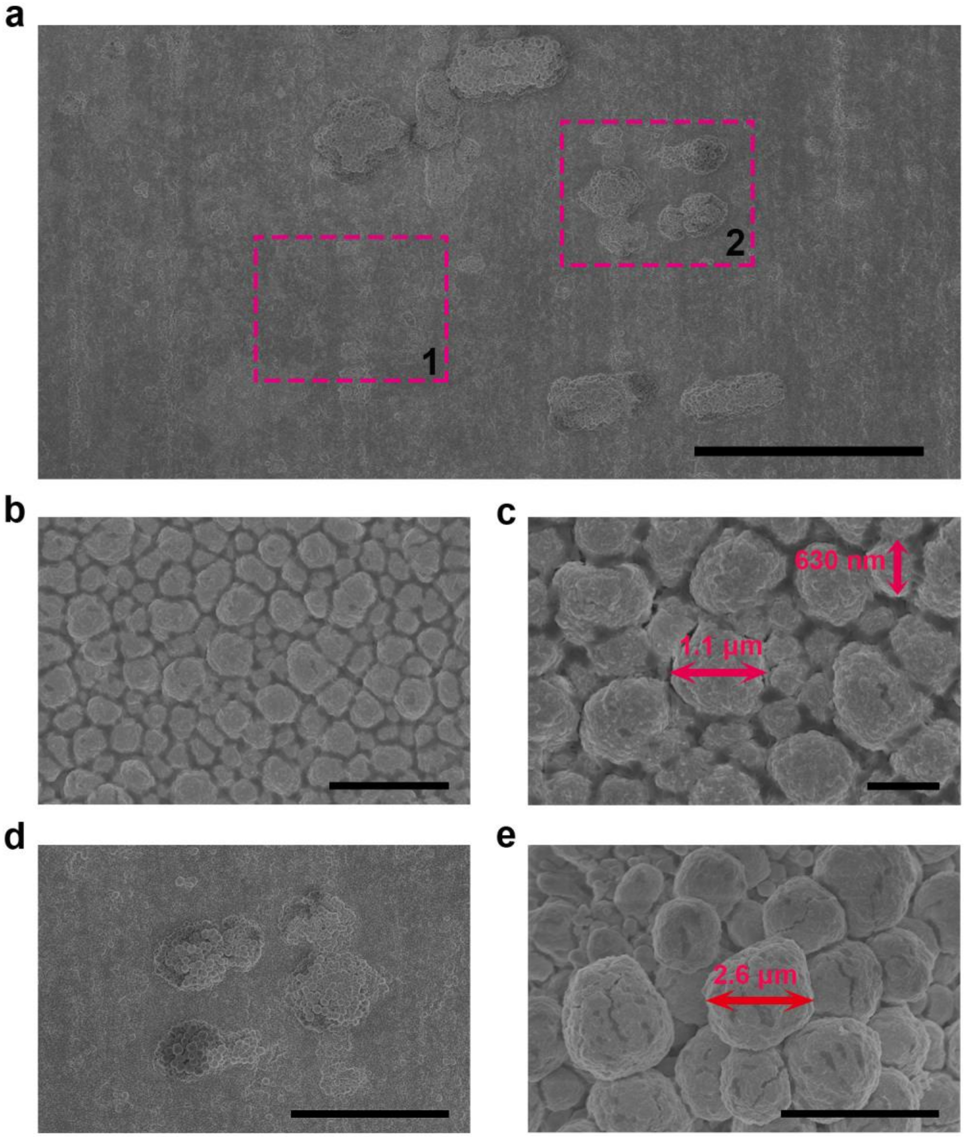

Supplementary Figure 13 | Morphology of Li deposits after galvanostatic plating for one time in $LiPF_6$-EC/DEC electrolyte with 0.41 mg mL^{-1} nanodiamond. (**a**) SEM images of Li deposits. The enlarged view of (**b**) - (**c**) region 1 and (**d**) - (**e**) region 2 in (**a**). The current is 0.5 mA cm^{-2} and plating time is 6 h. The scale bars in (**a**) - (**e**) are 100, 3, 1, 40, and 4 μm, respectively.

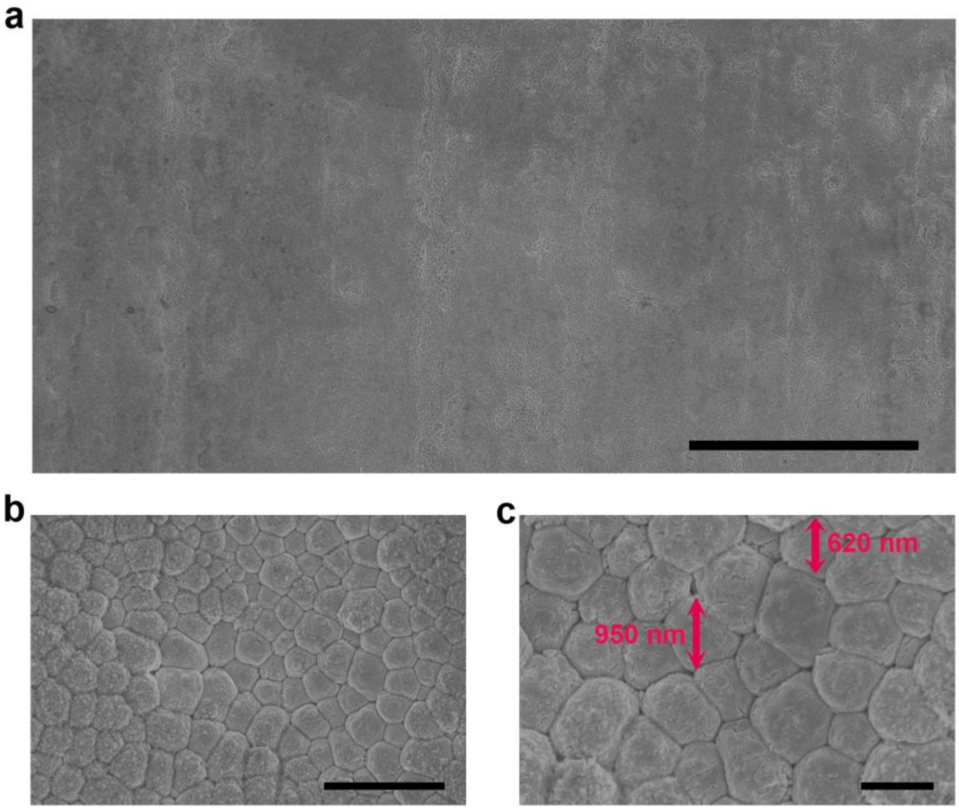

Supplementary Figure 14 | Morphology of Li deposits after galvanostatic plating for one time in $LiPF_6$-EC/DEC electrolyte with 4.1 mg mL^{-1} nanodiamond. SEM images of (**a**) - (**c**) Li deposits. The current is 0.5 mA cm^{-2} and plating time is 6 h. The scale bars in (**a**) - (**c**) are 100, 3, and 1 μm, respectively.

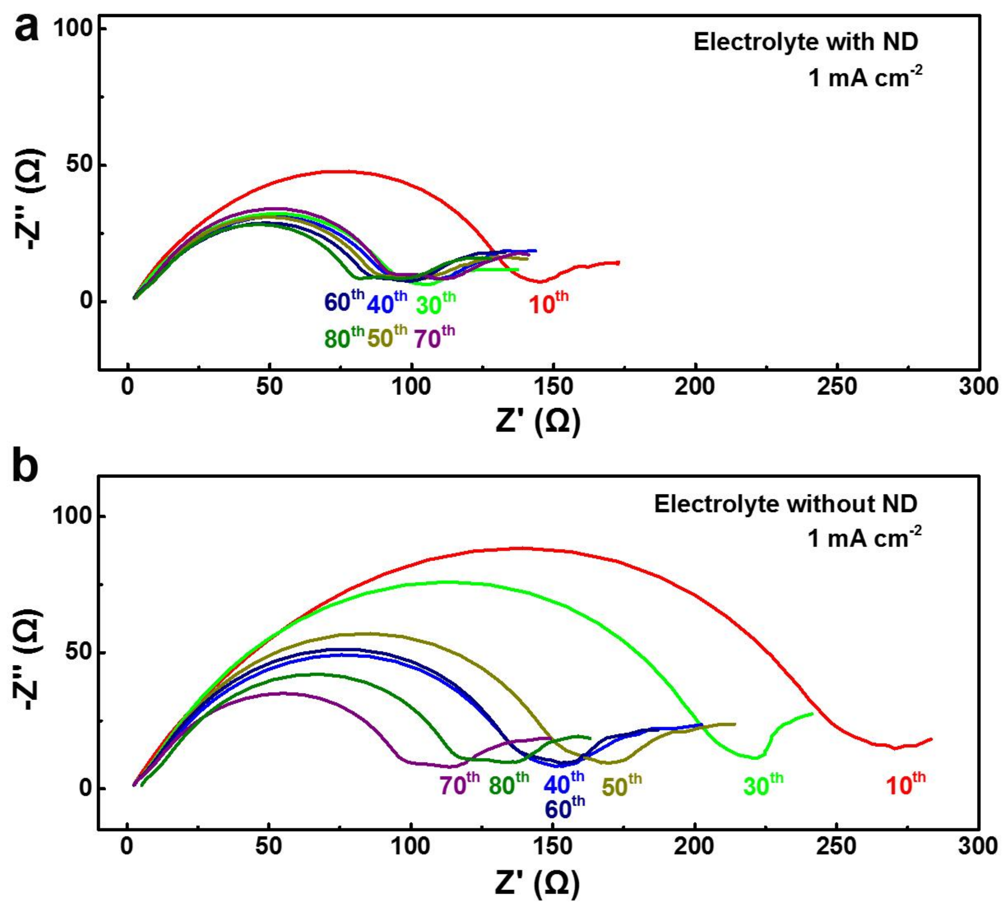

Supplementary Figure 15 | EIS of Li | Li cells at 1.0 mA cm^{-2}. (**a**) Electrolyte with nanodiamond. (**b**) Electrolyte without nanodiamond. The word 'ND' in the figure is the abbreviation of 'nanodiamond'.

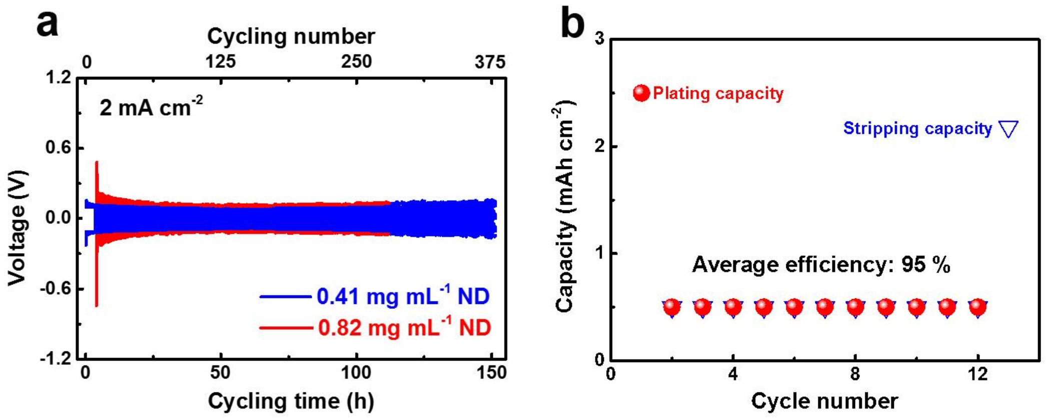

Supplementary Figure 16 | Electrochemical performance of nanodiamond electrolyte with different concentrations. (**a**) Charge-discharge curves of symmetrical Li | Li cells at 2 mA cm^{-2}. Each charge and discharge time is set as 12 min. (**b**) The plating and stripping capacities of cells in the 0.41 mg mL^{-1} nanodiamond electrolyte adopted to calculate the average Coulombic efficiency in 12 cycles. The word 'ND' in the figure is the abbreviation of 'nanodiamond'.

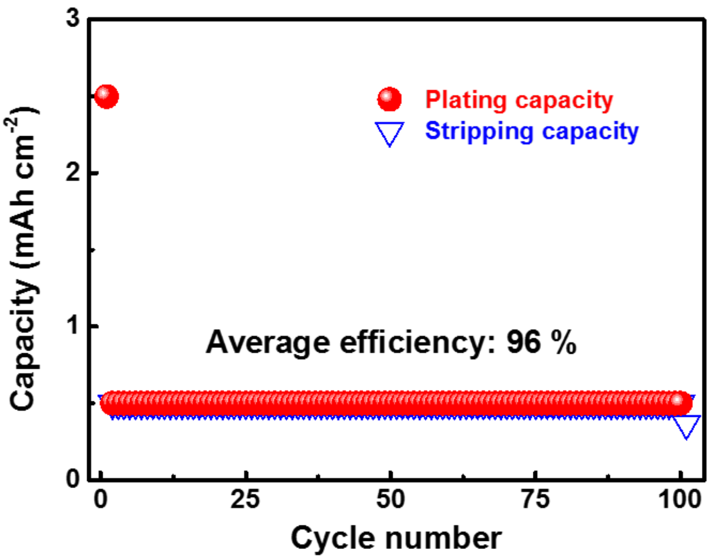

Supplementary Figure 17 | The plating and stripping capacities of cells in the 0.82 mg mL^{-1} nanodiamond electrolyte adopted to calculate the average Coulombic efficiency in 100 cycles.

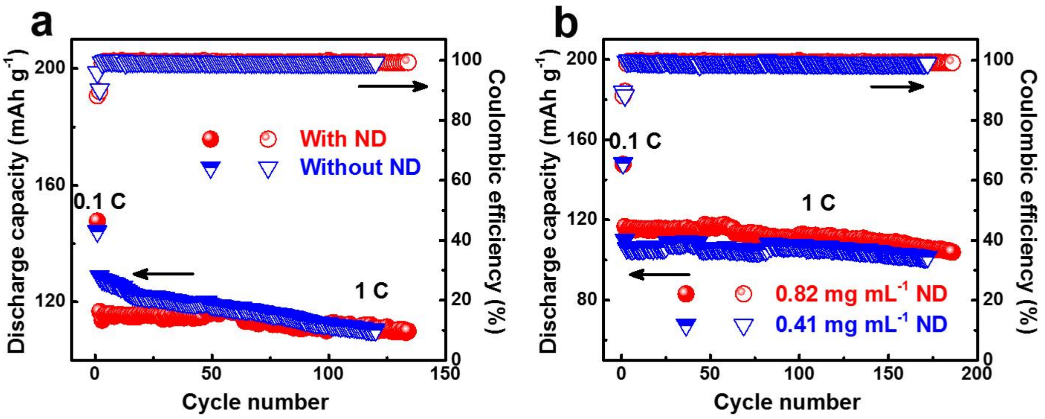

Supplementary Figure 18 | The cycling performance of Li | LFP cells at 1.0 C after an activation at 0.1 C (1.0 C = 180 mA g^{-1}). (**a**) The cycling stability of nanodiamond-contain electrolyte *vs.* nanodiamond-free electrolyte; (**b**) The cycling stability of 0.82 mg mL^{-1} *vs.* 0.41 mg mL^{-1} nanodiamond electrolyte. The word 'ND' in the figure is the abbreviation of 'nanodiamond'.

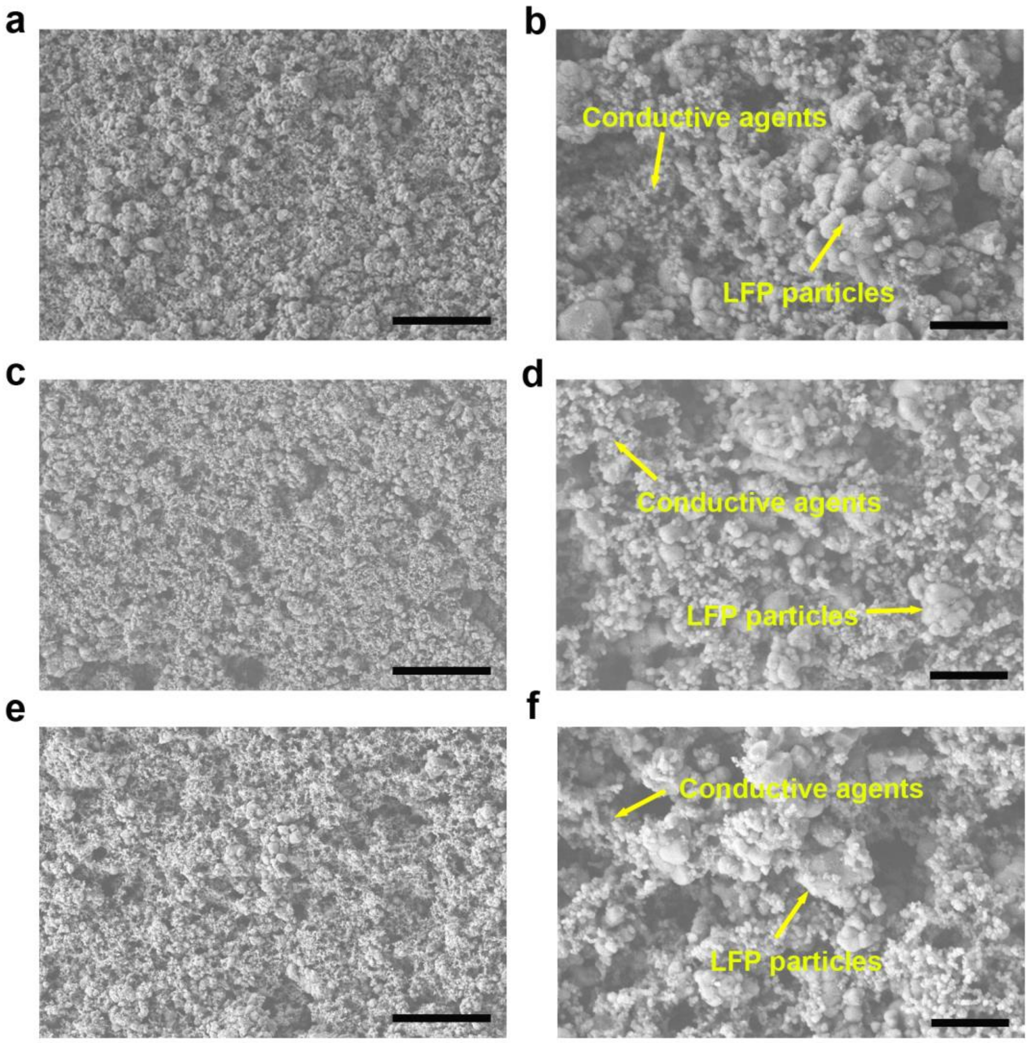

Supplementary Figure 19 | The morphology of LFP cathodes in Li | LFP cells. SEM images (**a**) – (**b**) before cycling, (**c**) – (**d**) after 5 cycles, (**e**) – (**f**) after 20 cycles. The scale bars in (**a**), (**c**), & (**e**) are 5 μm, in (**b**), (**d**), & (**f**) are 1 μm.

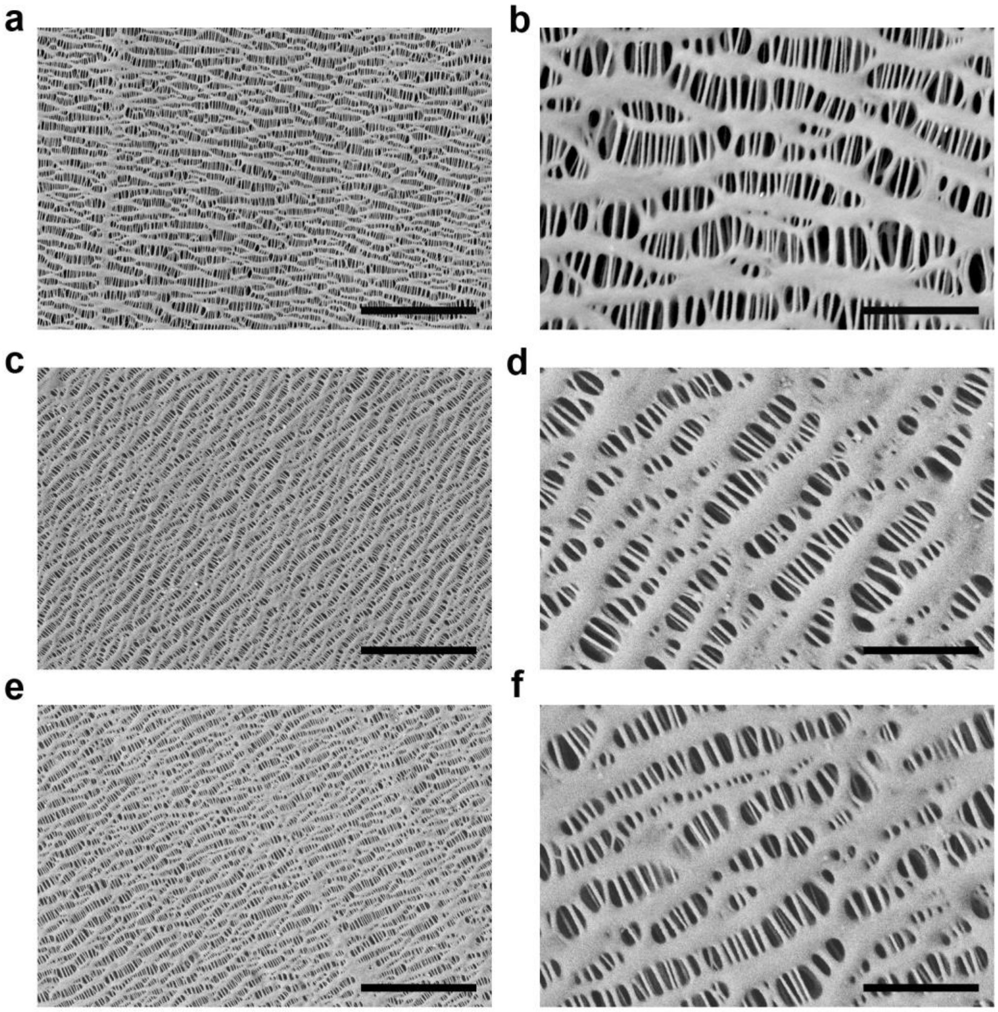

Supplementary Figure 20 | The morphology of separators in Li | LFP cells. SEM images (**a**) – (**b**) before cycling, (**c**) – (**d**) after 5 cycles, (**e**) – (**f**) after 20 cycles. The scale bars in (**a**), (**c**), & (**e**) are 5 μm, in (**b**), (**d**), & (**f**) are 1 μm.

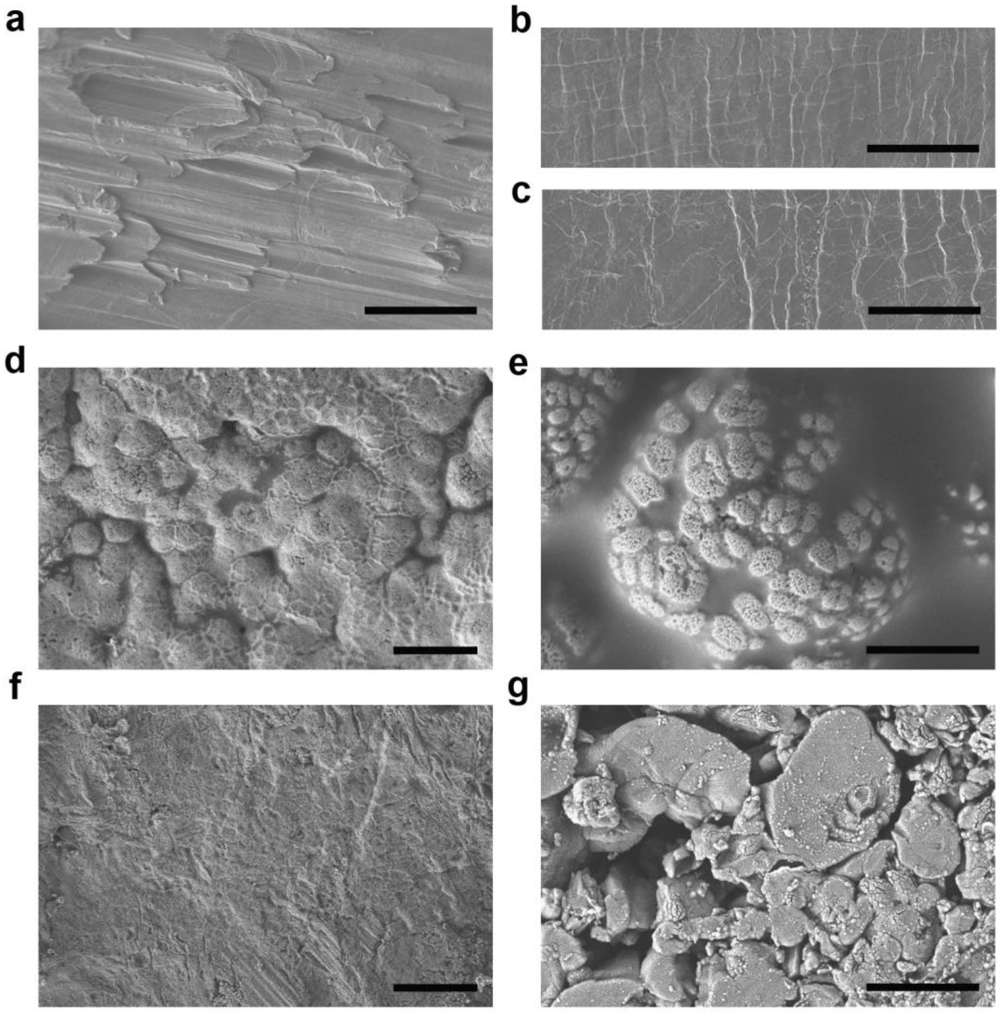

Supplementary Figure 21 | The morphology of Li anode. SEM images (**a**) – (**c**) before cycling, (**d**) – (**e**) after 5 cycles, (**f**) – (**g**) after 20 cycles in the Li | LFP cells. (**a**) is the as-received commercial Li foil. (**b**) and (**c**) are the pressed Li foils used in the coin-cell assembly. The scale bars in (**a**) & (**b**) are 100 μm, in (**c**) is 30 μm, in (**d**) & (**f**) are 10 μm, in (**e**) & (**g**) are 3 μm.

Supplementary Notes

Supplementary Note 1

In the Scherrer Equation, as θ decreases and β increases, D decreases. The XRD pattern of Li deposits in the nanodiamond electrolyte indicates a smaller θ and larger β, thus the Li deposit in the nanodiamond-containing electrolyte has a smaller grain size (D) than that in neat electrolyte.

$D=K\lambda/\beta\cos\theta$

where D is grain size, K is Scherrer constant (K= 0.89), λ is the X-ray wavelength, β is the full width at half the maximum intensity (FWHM), θ is the Bragg angle.

Supplementary Note 2

From the surface layer to Li metal, the signal of Cu rises. This is because Cu exists in the current collector and there is no Cu in the surface Li layer. F is present in the SEI layer due to $LiPF_6$ composition. Therefore, F herein is adopted as the indicator of the surface SEI film. Carbon can be introduced by the solvent decomposition and nanodiamond co-deposits. In nanodiamond-free electrolyte, the carbon content correlates with that of F, indicating the presence of the SEI layer. In the nanodiamond-containing electrolyte, F content decreases, while C content remains unchanged. If there were only decomposition products and no nanodiamond co-deposit, the signal of carbon would decrease. These results clearly confirm that nanodiamond co-deposits with Li.
